# Parental Meta-Emotion Philosophy and Emotion Coaching in Families of Children and Adolescents with an Anxiety Disorder

**DOI:** 10.1007/s10802-016-0180-6

**Published:** 2016-07-02

**Authors:** Katherine E. Hurrell, Frances L. Houwing, Jennifer L. Hudson

**Affiliations:** Centre for Emotional Health, Department of Psychology, Macquarie University, Sydney, Australia

**Keywords:** Childhood anxiety, Emotion regulation, Parenting, Emotion socialisation

## Abstract

Using a multi-method approach, this study examined differences in parental meta-emotional philosophy (including, parental emotional awareness and emotion coaching) for families with anxiety disordered (AD; *n* = 74) and non-AD (*n* = 35) children (aged 7 to 15). Further, it was investigated whether children’s emotion regulation (ER) varied across the AD and non-AD groups. Parent(s) were interviewed about their awareness of emotions and emotion coaching; completed a battery of questionnaires that included a measure assessing children’s emotion regulation; and engaged in a parent-child discussion task. Results indicated that compared to parents of non-AD youth, parents of AD youth were less aware of their own emotions and their children’s emotions, and these results varied by emotion type. Parents of AD youth engaged in significantly less emotion coaching than parents of non-AD youth. AD youth were identified as having significantly greater difficulty regulating their emotions when compared to non-AD youth. Implications for the role of parental meta-emotional philosophy and AD youth’s emotion regulation are discussed.

Emotional regulation (ER) deficits have been identified as risk factors for poor socioemotional adjustment and increased symptomatology (e.g., Cicchetti et al. 1995; Zeman et al. 2002). Even though non-clinical youth can show ER deficits, clinically anxious youth have been identified as displaying significantly more emotional competence deficits than non-clinical youth (e.g., Suveg et al. 2008; Suveg and Zeman 2004). These deficits include more difficulty regulating negative emotions (Hurrell et al. 2015; Suveg and Zeman 2004), lower confidence and knowledge about how to modify emotional states (Southam-Gerow and Kendall 2000; Suveg and Zeman 2004), and parents report that anxious youth are generally more emotionally labile and negative (Hurrell et al. 2015; Suveg and Zeman 2004). Moreover, when anxious youth with poor ER are compared to anxious youth who do not have poor ER, greater impairments in social functioning and more difficulties with several mood states are apparent (e.g., Kerns et al. 2014). Among non-clinical samples, researchers have established that several parenting factors are related to ER in children (Gottman et al. 1996). In particular, parents’ meta-emotion has been identified as impacting children’s ER (Gottman et al. 1996). Parental meta-emotion is defined by Gottman et al. (1996) as the feelings and thoughts that one has about emotion. Further, Gottman et al. (1996) state that parents’ meta-emotion philosophy refers to the organised set of thoughts and feelings that parents have about their children’s emotions and their own emotions. Although researchers have examined the relationship between parents’ meta-emotions and poor ER in non-clinical youth (Gottman et al. 1996), to date, researchers have not examined whether parents’ meta-emotions relate to poor ER in clinically anxious youth. This study, therefore, aims to examine whether parents’ philosophies towards meta-emotion is related to ER in clinically anxious youth. In this study, families of clinically anxious youth and non-clinically anxious youth will be compared so that differences in parental meta-emotion and child ER can be identified.

## Parental Meta-Emotion Philosophy

Gottman et al. (1996) asserted that parents’ philosophies towards meta-emotion can be assessed by examining parents’ awareness towards emotions in themselves and their children, their views about whether children’s negative emotions provide an opportunity for intimacy and teaching, whether parents tend to validate and label children’s emotions, and whether parents help children to problem solve in situations that result in negative child emotions. When examining these areas, the empirical evidence suggests that, in general, parents seem to hold either an emotion coaching or emotion dismissing philosophy (Gottman et al. 1997). Parents high in an emotion coaching philosophy view children’s negative emotions as opportunities for closeness and teaching (Gottman et al. 1996). They tend to be more aware of their own and their children’s emotions, are more likely to validate and label emotions, and often support their children with strategies to cope in emotionally arousing situations (Gottman et al. 1996, 1997). In contrast, parents with an emotion dismissing philosophy view negative emotions as harmful and tend to ignore, dismiss, or quickly attempt to alter negative emotions (Gottman et al. 1996, 1997).

Gottman et al. (1996) have found that parents’ meta-emotion philosophies influence the way in which children are socialised to experience and express emotions. Emotion coached children show evidence of good psychosocial adjustment and peer relations (Gottman et al. 1997). In comparison to children of parents with an emotion dismissing philosophy, emotion coached children tend to have better physiological and emotion regulation abilities, fewer externalising and internalising symptoms, higher self-esteem, less physiological stress, and higher levels of academic achievement (e.g., Shortt et al. 2010; Gottman et al. 1996). Children of parents who have an emotion coaching philosophy also tend to be more socially competent, engage in greater positive peer play, and have better social skills than children of parents with an emotion dismissing philosophy (e.g., Gottman et al. 1996).

### Parental Emotion Socialisation in Families of Anxious Children

In families of anxiety disordered (AD) children, researchers have examined emotion socialisation processes through parent-child emotion discussions (Hudson et al. 2008; Suveg et al. 2005; Suveg et al. 2008), parents’ reactions to children’s negative emotions (Hurrell et al. 2015), and by investigating related parenting styles, such as overprotection (e.g., Hudson and Rapee 2001; Wood 2006). To date, research has indicated that parents of AD children show less supportive responses to their children’s displays of not only anxiety, but also their expressions of other negative emotions (e.g., sadness and anger) when compared to non-anxiety disordered (non-AD) children. Parents of AD children cope with expression of negative child emotions (e.g., fear, sadness, and anger) by using maladaptive parenting strategies (e.g., overprotection, intrusiveness; e.g., Hudson et al. 2008). In particular, overprotective and controlling behaviours are observed more frequently in parents of AD children (Hudson and Rapee 2001; Siqueland et al. 1996), and parents of AD children believe these strategies help with dampening down, or preventing, child distress. Similarly, compared to parents of non-AD youth, parents of AD children tend to engage in more avoidant behaviours (e.g., change topics), provide less explanatory information about emotions, and interact in less pleasant ways when discussing emotional events with their children (Suveg et al. 2005; Suveg et al. 2008). Moreover, mothers of AD children self-report using significantly fewer emotion-focussed (e.g., comforting and soothing) and problem-focussed (e.g., problem-solving) strategies than mothers of non-AD children (Hurrell et al. 2015). Confirming mother’s self-reports, observers also have found that mothers of AD children show greater use of non-supportive parenting (e.g., criticism and talking over the child) and less use of supportive parenting (e.g., warmth) when responding to children’s negative emotions than mothers of non-AD children (Hudson et al. 2008).

AD youth, therefore, appear to be exposed to a qualitatively different family emotion environment than non-AD children. Thus, it is possible that this environment may accentuate ER vulnerabilities and subsequent internalising symptoms (Kerns et al. 2014; Zeman et al. 2002). In several theoretical models, poor ER is posited to underlie many forms of psychopathology (e.g., Gross and Muñoz 1995; Kring and Bachorowski 1999; Werner and Gross 2010). In relation to theoretical models of anxiety disorders, poor ER in regard to fear is considered to be a central feature in the aetiology, maintenance, and treatment of anxiety (e.g., Mennin et al. 2002). A family environment that promotes the development of poor ER across emotions is likely to place the child at greater risk for the development of anxiety and other psychopathology. Further, the role of parenting variables are frequently emphasised in theoretical models of child anxiety (e.g., Chorpita and Barlow 1998; McLeod et al. 2007). For instance, parental control and overprotection has been theorised to increase a child’s susceptibility to developing an AD by diminishing a child’s belief that they can cope on their own and restricting their opportunity to explore novel situations (e.g., Chorpita and Barlow 1998). Likewise, parents’ meta-emotion philosophies may guide parental behaviours and be associated with practices that do not adequately promote ER in children, such as providing little education on emotions, causes of emotions, and strategies to effectively manage emotions.

Parents of AD youth are likely to engage in poor emotion socialisation for a range of reasons, involving both child and parent factors (for a review see Morris et al. 2007). Common factors among parents who are found to be less responsive to their children’s emotions include higher stress levels (Nelson et al. 2009), more psychopathology (e.g., anxiety and depressive symptoms; Cummings et al. 2013; Suveg et al. 2005; Woodruff-Borden et al. 2002), and associated emotion deficits (e.g., difficulty tolerating negative emotions; Gross and Muñoz 1995; Hoffman et al. 2006). Parents of AD children often score high on measures of psychopathology, and rates of anxiety disorders, in particular, are substantial (Hettema et al. 2001; Last et al. 1987). AD children may, therefore, be inherently at a greater risk of receiving disrupted ER socialisation from their parents compared to non-AD children.

In addition to parent factors, child-specific factors, such as temperament and frequent emotional negativity (Kagan et al. 1989; Morris et al. 2002) appear to play a role with eliciting sub-optimal parenting. In particular, non-supportive parental reactions have been observed to follow children’s negative affective displays (e.g., Morelen and Suveg 2012; Hudson et al. 2009). Moreover, an interplay between child negative emotion and parenting has been found longitudinally, with child negativity predicting non-supportive parental reactions over time (Eisenberg et al. 1999).

### The Current Study and Hypotheses

Although there is growing literature on parents’ meta-emotion philosophies in non-clinical samples (Gottman et al. 1996), little is known about the meta-emotion philosophies of parents of AD children. By further understanding whether meta-emotion philosophies differ between families of AD and non-AD children, theoretical models of anxiety and associated treatments may be adapted and improved. In order to address these issues, the primary aim of this study was to examine whether meta-emotion philosophies differed for parents of AD and non-AD children. It was hypothesized that relative to parents of non-AD youth, parents of AD youth would: (a) self-report a meta-emotion philosophy that is lower on both emotion coaching and emotional awareness across three different emotion types (fear, sadness and anger); and (b) display fewer emotion coaching behaviours and more emotion dismissing behaviours during a parent-child discussion task. The second aim of this study was to investigate whether children’s ER varied between AD and non-AD children. It was hypothesised that compared to non-AD youth, AD youth would (c) have greater difficulty regulating a range of negative emotions, including fear, sadness, and anger; and (d) be more emotionally labile/negative. The final aim of this study was to use a multi-methods approach to examine parental meta-emotion constructs and child ER. This aim would be addressed using multiple methods, including observation and parental self-report (including, questionnaires and during an interview where children’s regulation of fear, sadness, and anger would be discussed). It was hypothesised that (e) there would be agreement across the multiple methods used in this study, and across multiple reporters (e.g., mothers and fathers).

## Method

### Participants

Two groups of children and their parent(s) participated in this study (*N* = 109). The AD group consisted of 43 girls (*M*_age_ = 9.65 years, *SD* = 2.16 years; range = 7 to 15 years) and 31 boys (*M*_age_ = 8.87 years, *SD* = 1.82 years; range = 7 to 15 years) who presented with their parents for treatment at our clinic in Australia. The non-AD group consisted of 21 girls (*M*_age_ = 9.52 years, *SD* = 2.29 years; range = 7 to 14 years) and 14 boys (*M*_age_ = 11.14 years, *SD* = 2.25 years; range = 7 to 14 years) who had never sought treatment from a mental health professional. Non-AD families were recruited from the community via advertisements in local sporting and recreational organisations, community noticeboards, online social media and local independent schools. Participating parents included 103 mothers (94.50 %) and 66 fathers (60.55 %). In the AD group, mean mother age was 41.97 years (*SD* = 5.36 years; range = 29 to 53 years), and mean father age was 44.78 years (*SD* = 5.58 years; range = 29 to 55 years). In the non-AD group, mean mother age was 42.71 years (*SD* = 4.81 years; range = 33 to 54 years), and mean father age was 46.23 years (*SD* = 5.80 years; range = 36 to 59 years). The participating families were primarily from a middle-class socioeconomic background, married, from two-parent households, and Caucasian (see Table 1 for demographic details). To ensure comparable socio-economic status, non-AD families were recruited from the same geographical area as AD families.

**Table 1:** Participant Demographic Characteristics

Sample	AD	Non-AD
Median Family Income ($AUD per annum)	83, 200–124, 799	83, 200–124, 799
Marital status (%)		
Married	81.09	80.01
Divorced/separated	4.05	8.57
Never married	1.35	5.71
Missing data	13.51	5.71
Family composition (%)		
Two-parent household	79.73	82.86
Single-parent	1.35	8.57
Step/blended	5.41	2.86
Missing data	13.51	5.71
Ethnicity (%)		
Australian	64.87	48.58
European	10.81	20.00
Other (e.g., Asian, African, Middle Eastern)	10.81	25.71
Missing data	13.51	5.71
Maternal Education (%)		
Year 10	5.41	2.87
Year 12	9.46	5.71
TAFE/Apprenticeship	4.05	8.57
Certificate/Diploma	29.73	20.00
Undergraduate Degree	20.27	17.14
Postgraduate Degree	31.08	40.00
Missing data	0.00	5.71
Paternal Education (%)		
Year 10	1.35	8.57
Year 12	6.76	5.71
TAFE/Apprenticeship	5.41	5.71
Certificate/Diploma	12.16	17.14
Undergraduate Degree	16.21	17.14
Postgraduate Degree	12.16	20.00
Missing data	45.95	25.71

$*AUD* Australian dollars, *TAFE* Technical and Further Education

Trained postgraduate clinical psychology students and Clinical Psychologists assessed the children using the semi-structured clinical interview, the Anxiety Disorders Interview Schedule for DSM-IV - Child and Parent Version (ADIS-IV-C/P: Silverman and Albano 1996). For the AD children (*n* = 74), this resulted in the following principal diagnoses: generalised anxiety disorder 48 %, social phobia 23 %, specific phobia 12 %, separation anxiety disorder 3 %, obsessive-compulsive disorder 3 % and panic disorder 3 %. An additional anxiety disorder diagnosis was given to 51 children in the AD group (25 % generalised anxiety disorder, 16 % social phobia, 17 % specific phobia and 15 % separation anxiety disorder). Further, 11 children in the AD group met criteria for an additional diagnosis other than anxiety: mood disorder (*n* = 4), attention deficit hyperactivity disorder (*n* = 1), sleep disorder (*n* = 5) and oppositional defiant disorder (*n* = 1). Children in the non-AD group did not meet diagnostic criteria for a psychological disorder based on the ADIS-IV-C/P. In addition, non-AD children scored within the normative range on the Spence Child Anxiety Scale – Child and Parent Versions (Spence 1998). No children were excluded from the non-AD group.

### Measures

#### Symptomatology

##### Child Anxiety Diagnoses

The Anxiety Disorders Interview Schedule for DSM-IV - child and parent versions (ADIS-IV-C/P; Silverman and Albano 1996) consists of child and parent semi-structured clinical interviews that makes diagnoses based on the criteria set out in the fourth edition of the Diagnostic and Statistical Manual of Mental Disorders (DSM-IV, American Psychiatric Association 1994). Children were assigned a diagnosis if either the parent or child reported that symptoms were causing significant interference in functioning, and if a Clinical Severity Rating (CSR) of 4 or more was assigned (in accordance with the clinician’s manual of the ADIS-IV; Silverman and Albano 1996). The ADIS-IV-C/P has demonstrated good inter-rater and test-retest reliability (e.g., Silverman and Albano 1996). Further, research from our clinic has demonstrated excellent reliability for the ADIS with interrater agreement of kappa =1.00 for an overall anxiety disorder diagnosis, and between Kappa = .80 and .93 for specific anxiety diagnoses (Lyneham et al. 2007).

##### Child Anxiety Symptoms

The Spence Children’s Anxiety Scale - parent report (SCAS; Spence 1998) is a 38-item measure of anxiety symptoms on six subscales: Generalised Anxiety Disorder; Obsessive-Compulsive Disorder; Specific Phobia; Panic and Agoraphobia; Separation Anxiety; and Social Anxiety. The measure contains an additional six positive filler items to reduce negative response bias. Respondents indicate the frequency with which each symptom occurs on a 4-point scale from 0 (*never*) to 3 (*always*). Sound psychometric properties have been reported, including adequate test-retest reliability, high internal consistency, and high concurrent validity (e.g., Spence 1998). In this study, Cronbach’s alpha for the total SCAS score was .94 for mothers’ reports and .67 for fathers’ reports.

##### Parent Self-Reported Psychopathology Symptoms

The Depression Anxiety Stress Scales-21 (DASS-21; Lovibond and Lovibond 1995) was included as a measure of parental depression, anxiety, and stress. Parents rated each of the 21 items using a 4-point Likert scale from 0 (*not at all*) to 3 (*most of the time*). The DASS-21 has been assessed as a reliable and valid instrument in both community and clinical samples, with high internal consistency, and good convergent and discriminant validity (e.g., Henry and Crawford 2005). For the current study, Cronbach’s alpha for the respective depression, anxiety and stress symptom sub-scales were .89, .71 and .83 for mothers, and .91, .80 and .80 for fathers*.*

#### Parents’ Self-Reported Meta-Emotion

The meta-emotion interview (MEI-revised; Katz and Gottman 1999) is a semi-structured interview that assesses parents’ awareness of their own emotions, parents’ awareness of child emotions, parents’ emotion coaching, and child ER. In each of these areas, three emotions were coded, namely fear, sadness, and anger. The MEI-revised was coded using the Meta-Emotion Coding System (as described by Katz et al. 1994). The first author was comprehensively trained in the administration and coding of the interviews by resources provided from the laboratory of Katz and Gottman (University of Washington, Seattle, U.S.A.). A second coder who was blind to group status, and the study hypotheses, conducted reliability coding across all three emotions for 20 % of the interviews. All ICCs found in this study are comparable to reliability reported in previous studies (e.g., Gottman et al. 1997). Psychometric properties of the MEI scales have yielded adequate internal consistency and construct validity (e.g., Gottman et al. 1996, 1997; Lagacé-Séguin and Coplan 2005).

##### Parents’ Awareness of Own Emotions

This area was assessed by rating parents’ ability to identify, discuss and distinguish emotions in themselves (e.g., “What is it like for you to be angry?”). Higher scores indicated more parental awareness of their own emotions. Analyses generated an intra-class correlation (ICC) of .90, *p* < .01, and Cronbach’s alpha for the three coded emotions ranged between .70 to .80.

##### Parents’ Awareness of Child Emotions

The second area was assessed by rating the parent’s ability to identify, discuss and distinguish emotions in his/her child (e.g., “What is like for your child to be angry?”). Higher scores indicated more parental awareness of child’s emotions. The ICC was found to be .87, *p* < .01, and Cronbach’s alpha ranged between .70 to .77.

##### Parents’ Emotion Coaching

The third area was rated according to the degree of involvement, interest and knowledge parents reported with regard to his/her child’s emotional experiences, respect towards their child’s emotions, sharing of emotional experiences with their child, and thought and energy given to what his/her child knows about emotions. For example, participants were asked, “What do you do to help your child with this emotion?”. Higher scores indicated parents reported engaging in more emotion coaching. Analyses showed that the ICC for this area was .89, *p* < .01, and Cronbach’s alpha ranged between .68 to .78. Although Cronbach’s alpha for this area is relatively weak, these estimates are comparable to the internal consistency statistics reported in previous research (e.g., Katz et al. 2014). As the removal of a subscale purely on the basis of a low Cronbach’s alpha has been criticised in the psychometric literature (e.g., Kline 2000; McCrae et al. 2011), this sub-scale was retained.

#### Observed Parental Emotion Coaching and Dismissing Behaviours

The conflict discourse task (Baker et al. 2011) asked parent-child dyads to discuss, for three-minutes, a current or recent topic that had been causing some conflict at home, and to work towards a resolution. This task aimed to elicit negative emotions naturalistically for the purpose of observing child emotion regulation and parental coaching behaviours. All variables were coded on a 5-point Likert scale ranging from 1 (*none*) to 5 (*high*). A second coder coded 20 % of the interaction tasks to establish ICCs. In this study, four areas were of interest and were coded using Baker et al.’ (2011) coding system.

##### Observed Parents’ Emotion Coaching Behaviours

Five coding items were used to assess parents’ emotion coaching, specifically the degree of structuring that the parent provides (teaching, reflecting and problem-solving to facilitate emotion understanding), level of sensitivity and acceptance of the child, validation and encouragement shown towards the child, parents’ enthusiasm and interest for the task, and the degree of parental intimacy, warmth and affection displayed during the interaction. The single rater ICC for this sub-scale was .87, *p* < .01, which is comparable to reliability reported in observations studies on emotion socialisation research with anxious samples (e.g., Suveg et al. 2005, 2008). Cronbach’s alpha was found to be .78, which is comparable to Baker et al. (2011).

##### Observed Parents’ Emotion Dismissing Behaviours

Parents’ emotion dismissing was assessed by examining parental derogation of the child, the degree of intrusiveness during the task, the amount of minimisation and/or discouragement of child’s emotion, and parental detachment and/or disinterest during the task. In addition to the four previously used items on the parents’ emotion dismissing sub-scale, a new item was developed for this study, and added to this sub-scale, to capture parents’ distress reactions. The single rater ICC for this sub-scale was .66, *p* < .05, which is comparable to previous research with anxious samples (e.g., Suveg et al. 2005, 2008). Similar to Baker et al.’ (2011) study, Cronbach’s alpha for this sub-scale was found to be .83.

#### Child Emotion Regulation

##### Parent-Reported Child ER and Lability/Negativity from Questionnaire Data

The emotion regulation checklist (ERC; Shields and Cicchetti 1997) is a 24-item parent-report measure where parents report on children’s typical methods of managing emotional experiences. This scale is rated on 4-point Likert scale from 1 (*never*) to 4 (*always*). The checklist has two subscales. For both subscales, Shields and Cicchetti (1997) report high internal consistency, strong construct validity with established measures of emotion regulation, strong discriminate validity and the ability to differentiate between well-regulated and poorly regulated groups.

- *Child emotion regulation*. This subscale measures appropriate emotional display, empathy and emotional self-awareness (e.g., “is empathetic towards others”). In this study, Cronbach’s alpha of .74 was found for mothers, and .76 for fathers.
- *Child emotional lability* / *negativity*. This subscale assesses a child’s lack of flexibility, mood lability and dysregulated negative affect (e.g., “exhibits wide mood swings”). This subscale had a Cronbach’s alpha of .86 for mothers, and .88 for fathers.

##### Parent-Reported Child ER from Interview Data

Data was also collected during the MEI-revised (Katz and Gottman 1999), as described above, to measure child ER according to parent-report. Child ER was assessed by rating the intensity, duration, and frequency of the child’s emotional experiences, competence to overcome emotions, and any concerns parents had in regard to the child’s experience, or expression of, emotion (e.g., “Is there anything s/he does to get over feeling sad?”). The ICC was .92, *p* < .01, and Cronbach’s alpha ranged from .81 to .89.

##### Observed Child ER

In order to obtain a measure of observed child emotion regulation, a new item was added to the conflict discourse task (Baker et al. 2011) examining children’s negative emotions. Children’s negative emotions were observed from children’s body language, facial expressions, emotional expressions, and tone of voice. This item was reverse coded to provide a measure of emotional regulation, and higher scores indicated that children appeared to be more emotionally regulated (i.e. showed less displays of negative emotions). The ICC for observed child ER was .66. As this scale consisted of only one item, Cronbach’s alpha could not be calculated.

### Procedure

After obtaining informed written consent from parents and adolescents and verbal assent from children (aged below 12 years), a trained clinical psychologist or intern clinical psychologist administered the ADIS-IV-C/P. The ADIS was administered to the available parent(s) in a single session; this meant that if both parents were available they attended the session together. During the parent interview children completed all self-report measures outlined above. During the child interview, parent(s) completed the self-report measures indicated above. If one parent was available, they completed the self-report measures from their perspective. If both parents were available, each parent completed their own set of self-report measures indicating their own perspective. One parent from each family was then administered the MEI-revised. Whenever possible, the researcher selected the parent for the MEI-revised based on their gender. This was done in an alternating fashion in order to balance the reporter’s gender distribution for the data collected on the MEI-revised. This resulted in 27 (45 % of MEI-revised interviews) father interviews and 33 (55 % of MEI-revised interviews) mother interviews. MEI-revised interviews were audio-recorded and were later coded. Following parental participation in the MEI-revised, parent and child participants were seated next to each other in a room and the experimenter presented instructions for the conflict discourse task. When both parents were present, each parent participated in a separate conflict discourse discussion with their child. The conflict discourse task was video recorded and later coded. All AD families were provided with treatment at the clinic. Non-AD families were reimbursed $50 for their time and travel expenses.

## Results

Whenever possible, available data for both mothers and fathers were used in analyses. In order to ensure that the Type-I error rate was not inflated due to the repeated-measures design (e.g., SCAS data was collected from both mothers and fathers who reported about the same child), and as the number of participants was not equal across the AD and non-AD groups, generalized estimating equations (GEEs) were used. GEEs account for correlations between responses in repeated measures designs, and do not require equal numbers of cases across groups (Burton et al. 1998; Hanley et al. 2003).

Results are presented in five sections. First, preliminary analyses investigating whether the AD and non-AD groups vary according to demographic variables or symptomatology are shown. Second, correlations between key study variables by parent gender are displayed. Third, analyses were conducted to determine whether parents’ meta-emotion varied according to group (AD or non-AD), parent gender, or emotion type (fear, sadness, anger). In order to cover the different aspects of parent meta-emotion, this resulted in three GEEs using data from the MEI-revised: parents’ awareness of their own emotions, parents’ awareness of their child’s emotion, and parents’ emotion coaching. Fourth, two GEEs were conducted to investigate whether parents’ emotion coaching behaviours and emotion dismissing behaviours varied across AD and non-AD group, or parent report. In the final section, four GEEs are conducted to examine whether child ER varies across group, parent report, or emotion type.

### Preliminary Analyses

There were no differences in mean age between AD and non-AD children, *t*(107) = 1.91, *p* = .06; AD, *M* = 9.32 years, *SD* = 2.05; non-AD, *M* = 10.17 years, *SD* = 2.38. Child gender did not differ between the AD and non-AD groups, *χ* ^2^(1.00, *N* = 109) = .02, *p* > .05 (Yates Continuity Correction used; AD = 41.9 % boys, 58.1 % girls; non-AD = 40.0 % boys, 60.0 % girls). Further chi-square analyses confirmed that the two groups did not significantly differ on maternal education, *p* = .616, paternal education, *p* = .705, ethnicity *p* = .062, income *p* = .410, maternal age *p* = .354, paternal age *p* = .174, family composition *p* = .176, and marital status *p* = .304.

In addition to the ADIS-IV-C/P results, child symptom measures were included in this study to provide additional support for the AD and non-AD group distinction. The mean scores for both child and parent measures of symptomatology for the AD and non-AD groups are presented in Table 2. A 2 parent report (mother, father) × 2 group (AD, non-AD) GEE was run on SCAS scores. AD children, *M* = 32.05, were found to have significantly higher SCAS scores than non-AD children, *M* = 8.60, *Wald χ* ^*2*^ (1) = 154.18, *p* < .001. Investigation of SCAS scores also showed that no children in the non-AD group scored above clinical cut offs. Further, a 2 parent gender (mother, father) × 2 group (AD, non-AD) x psychopathology type (depression, anxiety, stress) GEE was run on DASS-21 scores. There was a significant main effect for group, *Wald χ* ^*2*^ (1) = 5.73, *p* = .017. Parents in the AD group endorsed significantly more symptoms across the DASS-21 than parents in the non-AD group, *M* = 2.79. There was also a significant main effect for psychopathology type, *Wald χ* ^*2*^ (2) = 319.38, *p* < .005. Parents reported significantly more stress symptoms, *M* = 5.24, than depression symptoms, *M* = 2.92, *Wald χ* ^*2*^ (1) = 10.24, *p* < .001, and anxiety symptoms, *M* = 1.71, *Wald χ* ^*2*^ (1) = 17.64, *p* < .001. Parents also reported significantly more depression symptoms than anxiety symptoms, *Wald χ* ^*2*^ (1) = 7.26, *p* < .001. Moreover, there was a significant parent gender by psychopathology type interaction, *Wald χ* ^*2*^ (1) = 7.05, *p* = .029 (see Table 2 for mean values). Post-hoc tests revealed that fathers endorsed significantly more depression symptoms than mothers, *Wald χ* ^*2*^ (1) = 7.26, *p* < .001. There were no significant differences, however, between mothers’ and fathers’ endorsement of anxiety, *Wald χ* ^*2*^ (1) = 0.21, *p* = .833, and stress, *Wald χ* ^*2*^ (1) = 0.16, *p* = .871 symptoms.

**Table 2:** Means and Standard Errors for Study Measures (except the MEI-revised subscales, which are presented in Table 3)

Measure	AD (Mothers *n* = 70; Fathers *n* = 40)		Non-AD (Mothers *n* = 33; Fathers *n* = 26)	
Spence Child Anxiety Scale	*M*	*SE*	*M*	*SE*
Mother report	33.27	1.76	7.71	5.83
Father report	30.82	2.14	9.49	1.17
DASS-21	AD (Mothers *n* = 70; Fathers *n* = 40)		Non-AD (Mothers *n* = 33; Fathers *n* = 26)	
Mother self-report				
Depression	2.73	0.40	2.04	0.33
Anxiety	2.04	0.33	1.43	0.23
Stress	5.74	0.45	4.64	0.49
Father self-report				
Depression	4.11	0.66	2.82	0.55
Anxiety	2.11	0.47	1.24	0.31
Stress	6.01	0.58	4.55	0.60
Observed Emotion Coaching	AD (Mothers *n* = 28; Fathers *n* = 16)		Non-AD (Mothers *n* = 22; Fathers *n* = 14)	
	*M*	*SE*	*M*	*SE*
Mother report	13.93	0.92	18.73	0.81
Father report	13.04	1.03	18.14	1.33
Observed Emotion Dismissing	AD (Mothers *n* = 28; Fathers *n* = 16)		Non-AD (Mothers *n* = 22; Fathers *n* = 14)	
	*M*	*SE*	*M*	*SE*
Mother report	11.86	0.52	7.91	0.52
Father report	13.47	0.85	8.58	0.88
Child Emotion Regulation from ERC Questionnaire	AD (Mothers *n* = 39; Fathers *n* = 48)		Non-AD (Mothers *n* = 33; Fathers *n* = 27)	
	*M*	*SE*	*M*	*SE*
Mother report	24.94	0.57	28.67	0.43
Father report	24.33	0.51	27.30	0.50
Child Lability/Negativity from ERC Questionnaire	AD (Mothers *n* = 61; Fathers *n* = 48)		Non-AD (Mothers *n* = 33; Fathers *n* = 27)	
	*M*	*SE*	*M*	*SE*
Mother report	30.90	0.79	22.89	0.65
Father report	30.22	0.64	27.20	0.59
Observed Child Emotion Regulation	AD (Mothers *n* = 28; Fathers *n* = 16)		Non-AD (Mothers *n* = 22; Fathers *n* = 14)	
	*M*	*SE*	*M*	*SE*
Mother report	3.12	0.19	4.49	0.15
Father report	3.01	0.29	4.38	0.14

*DASS*-21 Depression Anxiety Stress Scales; *ERC* Emotion Regulation Checklist

### Correlations

See Table 4 for correlations between the variables of interest by parent gender.

### Effects of Parent Gender, Group, and Emotion Type on Parents’ Meta-Emotion

GEEs examining 3 emotion type (fear, sadness, anger) × 2 parent gender (mother, father) × 2 group (AD, non-AD) were run on the three parent meta-emotion subscales of the MEI-revised. Table 3 displays the overall means and standard errors for these subscales. Emotion type was a within-subjects factor, and parent gender was a between-subjects factor. As only one parent from each family was administered the MEI-revised, analyses included parent gender as a between-subjects factor to assess whether generally mothers differed to fathers in their meta-emotion philosophies.

**Table 3:** Means and Standard Errors for the Meta Emotion Interview variables, across the Emotions of Anger, Sadness, and Fear

	AD (*n* = 29)		Non-AD (*n* = 22)	
Variable	*M*	*SE*	*M*	*SE*
Parents’ awareness of own emotions				
*Fear*	15.93	0.45	18.77	0.33
*Sadness*	16.97	0.44	18.97	0.32
*Anger*	18.03	0.37	19.47	0.18
Parents’ awareness of child emotions				
*Fear*	16.26	0.46	19.34	0.20
*Sadness*	16.61	0.46	19.20	0.26
*Anger*	17.47	0.41	19.13	0.21
Parents’ emotion coaching				
*Fear*	17.79	0.49	24.14	0.34
*Sadness*	18.21	0.58	23.71	0.37
*Anger*	16.34	0.45	22.72	0.45
Parent-reported child ER from interview data				
*Fear*	11.48	0.53	19.01	0.33
*Sadness*	12.94	0.59	18.90	0.23
*Anger*	17.84	0.54	21.27	0.40

**Table 4:** Correlations among study variables by parent gender

Variables	1	2	3	4	5	6	7	8	9	10
1. Spence Child Anxiety Scale	-	−.31	.10	−.52	.13	−.06	−.30	.20	−.71*	−.44
2. Parents’ awareness of own emotions (MEI)	−.21	-	.86***	.69***	−.03	.49	−.30	−.04	.23	−.98**
3. Parents’ awareness of child emotions (MEI)	−.18	.76***	-	.86***	.35	−.35	−.16	−.36	.43	−.74
4. Parents’ emotion coaching (MEI)	−.34	.61***	.82***	-	.63	−.59	.30	−.46	.79***	−.46
5. Observed Emotion Coaching	−.50**	.63**	.76***	.88***	-	−.89***	.36	−.11	.79	.29
6. Observed Emotion Dismissing	.46**	−.74***	−.78***	−.87***	−.86***	-	−.34	.09	−.73	−.53
7. Child Emotion Regulation (ERC)	−.55***	.41*	.47**	.55**	.55***	−.54***	-	−.20	.63*	−.26
8. Child Lability/Negativity (ERC)	.60***	−.39*	−.49**	−.60***	−.56***	.54***	−.73***	-	−.56	.27
9. Parent-reported child ER (MEI)	−.57***	.37*	.46**	.73***	.65**	−.76**	.54**	−.54**	-	−.16
10. Observed Child Emotion Regulation	−.57***	.63**	.74***	.73***	.73***	−.80***	.50**	−.55***	.72**	-

Correlations in the lower left of the table are for mothers and those in the upper right are for fathers. *MEI* Meta Emotion Interview; *ERC* Emotion Regulation Checklist. ^***^ *p* < .001, ^**^ *p* < .01 ^*^ *p* < .05

#### Parent’s Awareness of Own Emotions

There was a significant main effect for group, *Wald χ* ^*2*^ (1) = 24.03, *p* < .001 and for emotion type, *Wald χ* ^*2*^ (2) = 29.73, *p* < .001. These main effects were subsumed by a significant group by emotion type interaction, *Wald χ* ^*2*^ (2) = 7.93, *p* = .019. For the AD group, there was a significant difference between parents’ awareness for all three emotions, *M*_fear_ = 15.93, *M*_sadness_ = 16.97, *M*_anger_ = 18.03, *Wald χ* ^*2*^ (2) = 24.75, *p* < .001. For the non-AD group, there was a significant difference between parents’ awareness of fear and anger, *M*_fear_ = 18.77, *M*_anger_ = 19.47, *Wald χ* ^*2*^ (1) = 2.51, *p* = .012, and sadness and anger, *M*_sadness_ = 18.97, *Wald χ* ^*2*^ (1) = 2.03, *p* = .042. For the non-AD group, there was no significant difference, however, between parents’ awareness of fear and sadness, *Wald χ* ^*2*^ (1) = 0.80, *p* = .424.

#### Parent’s Awareness of Child’s Emotions

A significant main effect was found for group, *Wald χ* ^*2*^ (1) = 40.50, *p* < .001. Although the main effect for emotion type was not significant, *Wald χ* ^*2*^ (2) = 4.23, *p* = .119, there was a significant emotion type by group interaction, *Wald χ* ^*2*^ (2) = 8.53, *p* = .014. Parents of an AD child reported that they were significantly less likely to be aware of their child’s feelings of fear, *M* = 16.26, than their child’s feelings of anger, *M* = 17.47; *Wald χ* ^*2*^ (1) = 2.78, *p* = .005. This difference was not observed, however, for the non-AD group, *Wald χ* ^*2*^ (1) = 0.94, *p* = .350. Even though there was not a significant main effect for parent gender, *Wald χ* ^*2*^ (1) = 0.08, *p* = .775, there was a significant parent gender by group interaction, *Wald χ* ^*2*^ (1) = 5.02, *p* = .025. Post-hoc tests showed that non-AD mothers, *M* = 18.74, were significantly less likely to report being aware of their child’s feelings than non-AD fathers, *M* = 19.71, *Wald χ* ^*2*^ (1) = 6.55, *p* = .010. There was no significant difference between AD mothers, *M* = 17.16, and fathers, *M* = 16.40, regarding their awareness of their child’s feelings, *Wald χ* ^*2*^ (1) = 1.26, *p* = .261.

#### Parent’s Emotion Coaching

There was a significant main effect for group, *Wald χ* ^*2*^ (1) = 128.31, *p* < .001. Parents reported that they were significantly less likely to emotion coach AD children, *M* = 17.44, than non-AD children, *M* = 23.52. A significant main effect was also found for emotion type, *Wald χ* ^*2*^ (2) = 20.85, *p* < .001. Parents reported that they engaged in significantly less emotion coaching for anger, *M* = 19.53, than for fear, *M* = 20.96, *Wald χ* ^*2*^ (1) = 3.62, *p* < .001, or for sadness, *M* = 20.96, *Wald χ* ^*2*^ (1) = 4.33, *p* < .001. There was no significant difference between parents’ emotion coaching of fear and sadness, *Wald χ* ^*2*^ (1) = 0.02, *p* = .985.

### Observed Parental Emotion Coaching and Dismissing Behaviours

Two GEEs examining 2 parent gender (mother, father) × 2 group (AD, non-AD) were run on the emotion coaching and emotion dismissing subscales of the conflict discourse task. Parent gender was a within-subjects factor and group was a between-subjects factor.

#### Observed Parents’ Emotion Coaching

There was a significant main effect for group, *Wald χ* ^*2*^ (1) = 18.18, *p* < .001. AD parents, *M* = 13.49, were observed to be significantly less likely than non-AD parents, *M* = 18.44, to use emotion coaching.

#### Observed Parents’ Emotion Dismissing

There was a significant main effect for group, *Wald χ* ^*2*^ (1) = 32.87, *p* < .001. AD parents, *M* = 12.67, were observed to be significantly more likely than non-AD parents, *M* = 8.25, to dismiss emotions.

### Children’s Emotion Regulation

#### Parent-Reported Child ER and Lability/Negativity from Questionnaire Data

Two GEEs examining 2 parent gender (mother, father) × 2 group (AD, non-AD) were run on the parent-reported child ER data and emotion lability/negativity data from the ERC. Parent gender was a within-subjects factor and group was a between-subjects factor. Means and standard deviations for this scale are shown in Table 2.

#### Parent-Reported Child ER from Questionnaire Data

There was a significant main effect for group, *Wald χ* ^*2*^ (1) = 42.10, *p* < .001. Non-AD children, *M* = 27.98, were significantly more likely to be reported as displaying ER than AD children, *M* = 24.64. There was also a significant main effect for parent gender, *Wald χ* ^*2*^ (1) = 4.04, *p* = .044. Mothers, *M* = 26.80, were significantly more likely to report that their child displayed ER than were fathers, *M* = 25.82.

#### Parent-Reported Child Emotional Lability/Negativity from Questionnaire Data

There were significant main effects for group, *Wald χ* ^*2*^ (1) = 51.42, *p* < .001, and for parent gender, *Wald χ* ^*2*^ (1) = 10.47, *p* = .001. These main effects were subsumed by a significant two-way interaction between group and parent gender, *Wald χ* ^*2*^ (1) = 19.81, *p* < .001. Fathers of non-AD children, *M* = 27.20, were significantly more likely to report that their child was emotionally labile/negative than mothers of non-AD children, *M* = 22.89, *Wald χ* ^*2*^ (1) = 34.74, *p* < .001. For AD children, there was no significant difference between mothers, *M* = 30.90, and fathers, *M* = 30.22, reporting of their child’s emotional lability/negativity, *Wald χ* ^*2*^ (1) = 0.64, *p* = .423.

#### Parent-Reported Child’s ER from Interview Data

To investigate the parent-reported child’s ER data from the MEI-revised, a GEE was run to determine whether there were differences between the 3 emotion types (fear, sadness, anger) × 2 parent genders (mother, father) × 2 groups (AD, non-AD). Emotion type was a within-subjects factor. Parent gender and group were between-subjects factors. As highlighted earlier, only one parent from each family was administered the MEI-revised, so analyses included parent gender as a between-subjects factor to assess whether mothers generally differed to fathers in their meta-emotion philosophies. Table 3 includes the overall mean and standard error for this sub-scale. There was a significant main effect for group, *Wald χ* ^*2*^ (1) = 176.90, *p* < .001, and emotion type, *Wald χ* ^*2*^ (2) = 88.98, *p* < .001. These main effects were subsumed by an emotion type by group two-way interaction, *Wald χ* ^*2*^ (2) = 21.40, *p* < .001. AD children were significantly more likely to show emotion regulation for sadness than fear, *M*_*sadness*_ = 12.94, *M*_*fear*_ = 11.48, *Wald χ* ^*2*^ (1) = 2.90, *p* = .004, but there was no significant difference between non-AD children’s emotion regulation for sadness and fear, *M*_*sadness*_ = 18.90, *M*_*fear*_ = 19.01, *Wald χ* ^*2*^ (1) = 0.29, *p* = .769. Both AD, *M*_*anger*_ = 17.84; *Wald χ* ^*2*^ (1) = 8.92, *p* < .001, and non-AD children, *M*_*anger*_ = 21.27; *Wald χ* ^*2*^ (1) = 3.92, *p* < .001, were significantly more likely to show emotion regulation for anger than fear. Similarly, both the AD, *Wald χ* ^*2*^ (1) = 6.46, *p* < .001, and non-AD groups, *Wald χ* ^*2*^ (1) = 4.33, *p* < .001, were significantly more likely to show emotion regulation for anger than sadness. Although there was not a significant main effect for parent gender, *Wald χ* ^*2*^ (1) = 3.30, *p* = .069, a significant parent gender by group two-way interaction was found, *Wald χ* ^*2*^ (1) = 8.31, *p* = .004. Fathers of AD children, *M* = 15.08, were significantly more likely than mothers of AD children, *M* = 13.09, to report that their child was able to regulate his/her emotions, *Wald χ* ^*2*^ (1) = 2.52, *p* = .012. In the non-AD group, there was no significant difference between mothers, *M* = 19.95, and fathers, *M* = 19.50, reporting about children’s emotion regulation, *Wald χ* ^*2*^ (1) = 1.48, *p* = .139.

#### Observed Child ER

To investigate the observed child’s ER data from the conflict discourse task, a GEE was run to determine whether there were differences between 2 parent gender (mother, father) × 2 group (AD, non-AD). Parent gender was a within-subjects factor, and group was a between-subjects factor. There was a significant main effect for group, *Wald χ* ^*2*^ (1) = 35.13, *p* < .001. Non-AD children, *M* = 4.44, were observed to be significantly more emotionally regulated than AD children, *M* = 3.07.

## Discussion

A parental meta-emotion philosophy characterised by high levels of emotional awareness and emotion coaching has been related to positive socio-emotional outcomes in normative child populations (e.g., Gottman et al. 1996). To date, researchers have not examined the parental meta-emotion philosophies of parents of AD children. The current study was, therefore, the first to examine whether parents’ meta-emotion philosophies differed for parents of AD and non-AD children. Further, this study examined AD and non-AD children’s ER. In addressing these two aims, this study employed multiple methods and multiple informants.

As expected, parents of AD and non-AD children were significantly different in their meta-emotion philosophies. In particular, parents of AD children were significantly less likely to be aware of their own emotions, less likely to be aware of their child’s emotions, and less likely to engage in emotion coaching than parents of non-AD children. The current findings align with previous research that indicates that lower parental emotional awareness and less use of emotion coaching is associated with poorer socio-emotional outcomes in children (e.g., Gottman et al. 1996). These results may help researchers to understand the mechanisms through which parenting factors may contribute to the development or maintenance of childhood anxiety. For instance, parents who are less able to detect subtle emotions in themselves and their children may be less likely to communicate with children about emotions, and they may be less likely to offer adaptive assistance with ameliorating children’s distress. Indeed, it has been found that parents who are less aware of their own emotions are more inclined to model maladaptive emotional coping strategies (Taylor 2000). Moreover, this may provide one explanation for why AD children may be prone to receiving less optimal parenting (e.g., overprotectiveness and encouraging avoidance) when coping with anxiety-provoking situations and negative emotions (e.g., Barrett et al. 1996; Hudson and Rapee 2001).

The type of negative emotion further influenced parents’ meta-emotion philosophies. Specifically, parents of AD children were significantly less likely to be aware of their own feelings of fear than sadness. Parents of non-AD children, however, were equally aware of their own feelings of fear and sadness. This finding is consistent with research that suggests that parents of AD children tend to under-report their own anxiety (e.g., Kendall and Suveg 2006). Since the current data suggests that parents of AD children may not be as aware of their affective fear responses, it would be useful to examine in future research whether parents of AD children are aware of their behavioural responses to feared stimuli. Such results would be useful for informing treatments that aim to reduce parents’ modelling of fear-responses to AD children. In this study, it was also found that parents of AD youth reported being significantly less aware of their child’s feelings of fear than their child’s feelings of anger. Parents of non-AD youth, however, were found to be equally as aware of their child’s feelings of fear and anger. As the parents of AD children presented for treatment for anxiety, it is surprising that these parents have reported less awareness of their child’s fear-based emotions. It is possible that parents of AD children are not aware of the full extent to which their child experiences fear, or, possibly, the extent of their child’s fear is only apparent at higher levels of severity. Future research is needed to further investigate this area, as diminished parental awareness of fear may be an important factor in the development and maintenance of children’s anxiety problems. For example, reduced parental awareness may lead to insufficient or maladaptive parental management of children’s fear. In addition to these findings, parents in both groups were found to be less likely to emotion coach anger than fear or sadness. Although this finding was consistent across the AD and non-AD groups, it suggests that parents may find it more challenging to emotion coach anger than fear or sadness.

As expected, parents of AD youth were observed to show fewer emotion coaching behaviours and more emotion dismissing behaviours than parents of non-AD youth during the conflict discourse task. These results are consistent with research conducted by Suveg et al. (2008) where it was found that parents of AD youth engaged in few explanatory discussions of emotions. In particular, fathers engaged in few explanatory discussions for all emotions (e.g., happy, anxious, and angry), whereas mothers engaged in few explanatory discussions with sons regarding anxiety (Suveg et al. 2008). There was also a tendency for both mothers and fathers to discourage emotion discussions for anger (Suveg et al. 2008). In a study by Hudson et al. (2008), that examined dimensions of parents’ intrusive involvement and warmth, mothers of AD children were found to be significantly more intrusive when discussing negative emotions than mothers of non-AD children. Additionally, parents of AD children displayed lower levels of warmth than parents of non-AD children. When taken together, these findings suggest that emotion-socialisation processes may be awry in families of AD children, and that these processes may contribute to, or maintain, child anxiety. Evidence from previous research indicates that these types of parenting practices tend to predict emotion-related deficits in children, including poor emotion regulation (e.g., Fabes et al. 2001; Hooven et al. 1995; Ramsden and Hubbard 2002) and a higher risk for internalising and externalising problems (Zeman et al. 2002). Further study is needed, however, to investigate the direction of these effects, as it is possible that parental behaviours may be a reaction to child anxiety (e.g., Hudson et al. 2009).

Children’s emotion regulation was also found to vary according to group. As expected, independent observers and parents rated that AD children were significantly less likely to show emotion regulation than non-AD children. Further, as expected, AD children were rated by their parents as being more emotionally labile and negative than non-AD children. It was also found that the type of negative emotion further influenced parents’ perceptions of their child’s ability to regulate emotions. Both AD and non-AD groups were reported by their parents as having more difficulties with regulating sadness and fear in comparison to anger. Further, the AD group were reported by their parents as having the most difficulty in regulating their fear. When taken together, these findings provide further evidence for an emotion dysregulation model of anxiety (e.g., Mennin et al. 2005; Suveg et al. 2010). The current findings are consistent with research showing that AD youth have difficulties with managing negative emotions when compared to non-AD youth (Suveg and Zeman 2004; Hurrell et al. 2015). Moreover, the present findings are consistent with research by Suveg et al.’ (2008) where it was found that emotion dysregulation fully mediated the relationship between behavioural inhibition and high anxiety levels. In Suveg and colleagues’ study (2008), it was also shown that emotion dysregulation mediated the relationship between family emotional styles (emotional restrictiveness) and anxiety symptoms. Thus, low levels of emotional expressiveness in the family may contribute to the development of ER problems in children, as there are limited opportunities to explore and discuss emotions in the family context (see Denham et al. 1997).

Consistent with the hypothesis, there was generally agreement across the multiple measures used in this study. In particular, according to parent-report and observation, parents of AD children were less likely to have an emotion coaching philosophy than parents of non-AD children. There was also agreement across interview, questionnaire, and observational measures that AD children were less likely to regulate their emotions than non-AD children. Despite the agreement across measures, there was some disagreement between raters. In particular, mothers of AD and non-AD children were more likely than fathers to report on the questionnaire measure that their child showed emotion regulation behaviours. When interviewed, however, mothers and fathers of non-AD children were equally as likely to report that their child showed emotion regulation behaviours. When fathers of AD children were interviewed, they were significantly more likely than mothers to report that their child displayed emotion regulation behaviours. It was also found that mothers of non-AD children were less likely than fathers to report that their child was emotionally labile/negative. There was no difference, however, between mothers’ and fathers’ reporting of non-AD children’s emotional lability/negativity. These inconsistencies highlight the importance of considering different methods when investigating children’s emotion regulation behaviours. Moreover, these findings reinforce the need to consider the differences between mothers’ and fathers’ views of the same child.

Clinically, the findings from this study have important implications. Incorporating strategies that improve parental emotional awareness, parental emotion coaching, and children’s emotion regulation into treatment programs may enhance clinical outcomes for AD children. Although the current research highlights that in comparison to non-AD youth, AD youth have poorer emotion regulation and parents of AD youth are less likely to hold an emotion coaching meta-emotion philosophy, it must be noted that these findings may not be specific to AD children. Investigations of parents’ meta-emotion philosophies and youth’s emotion regulation in other clinical populations, has shown that adolescents with depression tend to experience fewer depression symptoms when their mothers are more accepting and expressive of their own emotions, and engage in emotion coaching (e.g., Katz and Hunter 2007). Further, findings consistently demonstrate an association between parents’ use of emotion coaching and better psychosocial adjustment in children with conduct problems (e.g., Dunsmore et al. 2013) and children at risk of abuse (e.g., Katz and Windecker-Nelson 2006). It is, therefore, possible that parents’ meta-emotion philosophy and children’s emotion regulation may be a transdiagnostic factor that may contribute to the onset and maintenance of several clinical presentations. In regard to treatment programs, research with other clinical groups has found that there are benefits to adding in treatment components that improve parental emotional awareness, parental emotion coaching, and children’s emotion regulation (e.g., Havinghurst et al. 2013). Such findings provide exciting avenues for future research and the ongoing development of treatment programs for AD youth and their parents. Future research could also benefit from determining the ideal content, context, and frequency of coaching responses when children express fear. For example, a parent may respond to a child’s anxious reassurance seeking in a way, that whilst understands and validates the child’s emotions, overly reassures the child’s cognitions thereby maintaining the child’s worry through reinforcing the child’s beliefs about their inability to cope on their own. It is important that emotion coaching delivers effective strategies in anxiety management.

This study is not without limitations. The sample was largely middle-class and Caucasian, which impacts the generalisability of the findings to other populations. In addition, the sample size was relatively small and there were unequal participant numbers among the groups. Generalised estimating equations were used to analyse the data to ensure that statistical assumptions about equal group sizes would not be violated. Despite this, it may be useful to replicate the current study with a larger and more diverse sample. In addition to being demographically diverse, it may be useful to examine a clinically diverse sample. This would be useful as it cannot be determined whether the current findings are specific to AD children or whether children with other clinical disorders may have ER deficits and parents with similar meta-emotion beliefs. In regards to measures, both interview and self-report measures are prone to social desirability biases, which could have influenced the findings. Whilst observational tasks tend to have more ecological validity, the laboratory setting is contrived and the contrived environment may have exerted an influence on participants’ expectations and behaviours. Moreover, the conflict discourse task only represents a ‘snapshot’ of parent-child interactions, which may not be representative of typical parent-child interactions outside of the laboratory. This limitation may have been further mitigated by providing parent-child dyads with the freedom to choose their own topic and by the experimenter leaving the room. Although child ER was assessed with both parental report and an observational task, future research may benefit from examining AD children’s self-report of their emotion regulation and comparing it to non-AD children’s self-report. Additionally, the focus of this research was on negative emotions so results may not be able to generalise to AD and non-AD children’s experience with positive emotions. Finally, this research was cross-sectional and, thus, causal conclusions cannot be drawn. Longitudinal research is required to determine the causal impact of parents’ meta-emotion philosophies on children’s ER, as well as whether children’s ER skills impact parents’ meta-emotion philosophies.

Despite these limitations, the present results contribute to the fields of child anxiety and parenting in several ways. First, the results have highlighted that parents of AD children are less likely than parents of non-AD children to have an emotion coaching philosophy when responding to their children’s emotions. This is a novel finding and may be important for clinical treatments and theoretical advances. Second, AD children were significantly less likely to show emotion regulation than non-AD children. Finally, there was generally agreement across the multiple methods of measurement used in this study. Future research on AD children should consider the role of parents’ meta-emotion philosophies when examining parenting practices and investigate how parents’ beliefs about meta-emotions may drive their emotion coaching behaviours and impact children’s socio-emotional functioning. It may also be worthwhile to extend this study by observing emotion coaching during family discussions that involve both positive and negative emotions.
